# Dynamic remodeling of the pancreas immune landscape in obesity

**DOI:** 10.1101/2025.07.14.664803

**Published:** 2025-07-18

**Authors:** Alexey Koshkin, Kranthi Kiran Kishore Tanagala, Anna Eichinger, Michael Chait, Aoife Young, Shanila Shakil, Junichi Yoshikawa, Yosuke Sakamoto, Steven B. Wells, Xiaojuan Chen, Boris Reizis, Donna L. Farber, Stuart P. Weisberg

## Abstract

Obesity is a known risk factor for diseases of the pancreas, including diabetes, pancreatic cancer and pancreatitis, but mechanisms remain unclear. To elucidate how obesity impacts pancreatic immune homeostasis, we performed spatial, transcriptomic and functional profiling of human pancreatic immune cells from obese and non-obese organ donors. Obesity was associated with higher density of tissue resident memory T-cells (TRM) in the exocrine pancreas which display high cytotoxic functions and aggregated around macrophages. Single cell sequencing of pancreatic macrophages revealed two main subsets - FOLR2 ^+^ CD11c ^−^ fetal-derived macrophages with pro-repair and immunoregulatory function and a FOLR2 ^−^ CD11c ^+^ monocyte-derived macrophages with greater T-cell interactions and pro-inflammatory function. In obesity, the pancreatic macrophage landscape shifts to lower predominance of FOLR2 ^+^ CD11c ^−^ macrophages and higher FOLR2 ^−^ CD11c ^+^ macrophages which interact selectively with the TRM and inflamed exocrine epithelium. Together, these results identify macrophage-T cell circuits and immune epithelial interactions that fuel chronic pancreatic inflammation in obesity – a potential unifying mechanism for obesity-related pancreatic diseases.

## Full Text Availability

The license terms selected by the author(s) for this preprint version do not permit archiving in PMC. The full text is available from the preprint server.

